# Phospho-specific antisera to monitor N-terminal autophosphorylation of cGMP-dependent protein kinase type I

**DOI:** 10.1186/2050-6511-14-S1-P74

**Published:** 2013-08-29

**Authors:** Raghavan Vallur, Hubert Kalbacher, Jana Krauß, Robert Feil

**Affiliations:** 1 Interfakultäres Institut für Biochemie, University of Tübingen, Tübingen, Germany; 2 German Center for Neurodegenerative Diseases (DZNE), Tübingen, Germany; 3 Graduate School of Cellular & Molecular Neuroscience, University of Tübingen, Tübingen, Germany

## Background

Although the cGMP-dependent protein kinase type I (cGKI) is an important mediator of cGMP signaling in many cells and tissues, its’ in vivo-biochemistry is not well understood. It has been shown that the purified enzyme can autophosphorylate multiple sites in its N-terminal region in the presence of ATP and cyclic nucleotides (cGMP and/or cAMP). N-terminal autophosphorylation might be involved in the activation of the kinase by cGMP in vitro, but it is not clear whether or not this also happens in intact cells [1].

## Results

To detect autophosphorylated cGKI in cells and tissues, we have generated polyclonal rabbit antisera against the major in vitro autophosphorylation sites of murine cGKI-alpha (Ser-50; Thr-58, Ser-72, and Thr-84) and cGKI-beta (Thr-56, Ser-63, and Ser-79). ELISAs with peptides containing the respective amino acids in their non-phosphorylated or phosphorylated form as well as Western blots with purified cGKI-alpha and cGKI-beta indicated that the antisera specifically recognized the autophosphorylated N-termini of these isoforms. The sensitivity of detection was comparable to a highly sensitive pan-cGKI antiserum. Interestingly, the addition of ATP (100 µM) alone was sufficient to induce autophosphorylation of the purified isozymes in vitro. Surprisingly, we were not able to detect phospho-cGKI species in intact fibroblasts and vascular smooth muscle cells, both under basal conditions as well as after induction of cGKI kinase activity (monitored as VASP phosphorylation) with cGMP-elevating compounds.

## Conclusion

We have generated phospho-specific antisera against the N-terminal regions of cGKI-alpha and cGKI-beta and could confirm the previously reported autophosphorylation of these isozymes in vitro. However, our results question the relevance of N-terminal autophosphorylation of cGKI in intact cells.
